# Status-neutral community-based multilevel intervention to address intersectional stigma and discrimination, and increase HIV testing, PrEP, and ART uptake among YGBMSM in Ghanaian Slums: A clustered randomized control trial protocol

**DOI:** 10.21203/rs.3.rs-4486078/v1

**Published:** 2024-05-30

**Authors:** Gamji Rabiu Abu-Ba’are, Kwasi Torpey, Chris Guure, LaRon E Nelson, Sangchoon Jeon, James McMahon, Natalie M Leblanc, Osman Wumpini Shamrock, Edem Yaw Zigah, Amos Apreku, Henry Delali Dakpui, George Rudolph Kofi Agbemedu, Francis Boakye, Prince Adu, Andrew Attisoe, Gideon Adjaka

**Affiliations:** Behavioral, Sexual, and Global Health Lab, School of Nursing, University of Rochester Medical Center, University of Rochester, New York, USA; Department of Population, Family and Reproductive Health, School of Public Health, University of Ghana, Accra, Ghana.; Department of Biostatistics, School of Public Health, University of Ghana, Legon-Accra, Ghana.; School of Nursing, Yale University, New Haven, Connecticut, USA.; School of Nursing, Yale University, New Haven, Connecticut, USA.; School of Nursing, University of Rochester Medical Center, University of Rochester, New York, USA.; School of Nursing, University of Rochester Medical Center, University of Rochester, New York, USA.; Behavioral, Sexual, and Global Health Lab, School of Nursing, University of Rochester Medical Center, University of Rochester, New York, USA; Behavioral, Sexual, and Global Health Lab, School of Nursing, University of Rochester, Rochester, New York, USA; Department of Population, Family and Reproductive Health, School of Public Health, University of Ghana, Accra, Ghana.; Behavioral, Sexual, and Global Health Lab, School of Nursing, University of Rochester Medical Center, University of Rochester, New York, USA.; Behavioral, Sexual, and Global Health Lab, School of Nursing, University of Rochester Medical Center, University of Rochester, New York, USA.; Priorities on Rights and Sexual Health, Accra, Ghana; Priorities on Rights and Sexual Health, Accra, Ghana; Priorities on Rights and Sexual Health, Accra, Ghana; Hope Alliance Foundation, Accra, Ghana

**Keywords:** HIV, Intersectional Stigma, Testing, Pre-Exposure Prophylaxis, Antiretroviral Therapy, Healthcare

## Abstract

**Background:**

While GBMSM constitute less than 2% of Ghana’s population, their HIV prevalence surpasses the national average by more than eightfold, emphasizing the critical need for targeted interventions to improve detection, care linkage, and reduce community transmission. This study seeks to increase HIV testing, Pre-Exposure Prophylaxis (PrEP), and Antiretroviral Therapy (ART) uptake (HPART) among YGBMSM through the adaptation of an evidence-based intervention (LAFIYA).

**Methodology:**

We will employ the ADAPTT-IT framework to adapt LAFIYA and evaluate its feasibility and effectiveness in addressing intersectional stigma and increasing HPART uptake among YGBMSM residing in Ghanaian slums. In aim 1, we will hold focus groups (n=5) and interviews (n=20) among YGBMSM and two FGDs among GBMSM-led organizations. At the HCF level, we will hold 6 FGDs and interviews (n=20) among nurses. In AIM 2, we will randomly assign 6 healthcare facilities (HCFs) to receive the LAFIYA (n=3) or wait-list control (n=3). Friend groups (cluster) of YGBMSM (N=240) will be assigned to receive LAFIYA (n=120) or a wait-list control (n=120). We will collect 3-, 6-, and 9-months post-intervention data among YGBMSM(n=240) and HCWs(n=300) to measure HPART adherence (primary outcomes), ISD reduction, HIV and status-neutral knowledge (secondary outcomes), and intervention acceptability, appropriateness, and feasibility (implementation outcomes).

**Conclusion:**

The intervention group will observe increased HPART adherence, reduced ISD, and enhanced HPART knowledge and efficacy relative to the wait-list control group. The findings will inform ISD reduction and HIV status-neutral implementation strategies – and place-based interventions that address access to HIV prevention and care among YGBMSM, slum and in different settings.

**Trail Registration:**

This study was registered on clinicalTrail.gov, with identifier number NCT06312514 on 03/14/2023. https://classic.clinicaltrials.gov/ct2/show/NCT06312514.

## Introduction

In Ghana, the HIV epidemic continues to pose significant public health challenges, particularly among gay, bisexual, and other men who have sex with men (GBMSM)^1–13^. Contributing to less than 2% of Ghana’s population, the HIV prevalence of this key population surpasses the national average by more than eightfold, emphasizing the need for targeted interventions to improve early detection, care linkage, and reduce community transmission^14–16^. Young GBMSM (YGBMSM) represent a vulnerable population that requires focused attention due to their increased HIV vulnerability^17–20^. YGBMSM aged 18 to 24 years comprise a sub-risk population within the broader GBMSM community, accounting for 63% of known HIV cases among GBMSM in Ghana^15^.

In addition to being young or GBMSM, living in urban areas, especially slums, can increase HIV vulnerability^21–23^. Slums house over 30% of Ghana’s urban residents, about 40% of our study area (Accra)^24,25^, and GBMSM in Accra have the highest HIV prevalence (44%) compared to nationwide GBMSM (18%)^26^. YGBMSM in slums remain at heightened vulnerability as slums remain documented high HIV-burdened areas due to structural issues (e.g., lack of access to care) and behavioral factors (e.g., transactional sex)^21–5,21,27–37^.

The advent of HIV self-testing (HIVST) and Pre-Exposure Prophylaxis (PrEP) alongside the established Antiretroviral Therapy (ART) represents significant milestones in the ongoing efforts to curb HIV incidence and enhance treatment adherence among diverse populations, including GBMSM^38–40^. HIVST facilitates repeated testing of self, partners, and clients^38^. The World Health Organization (WHO) recommends oral PrEP for persons with seronegative HIV tests at substantial and ongoing vulnerability to HIV. PrEP effectively prevents HIV acquisition but requires high adherence; adherence is the main predictor of effectiveness^39,40^. Studies in Ghana have reported low levels of knowledge but high acceptability of PrEP and HIVST among GBMSM^41–43^. Perspectives of policymakers in Ghana also indicate that HIVST is highly acceptable, but national guidelines and stakeholder consultations are necessary for successful implementation^44^.

Nonetheless, the uptake of HIV testing, PrEP, and ART (HPART) remains low among GBMSM in Ghana, with many researchers pointing to ISD as a key deterrent^42,45^. In Ghana, ISD towards GBMSM, particularly in the context of HIV, persists despite significant advancements in HIV awareness and advocacy^46,47^. GBMSM also face ISD due to legal, religious, and cultural antagonism, with 90% of Ghanaians against same-sex intercourse^16,48^. Existing evidence suggests a strong association between ISD and low HPART outcomes among this key population in the country^45,46,49–51^. At the intrapersonal level, GBMSM internalizes ISD experiences, develops guilt, shame, etc., and avoid HCFs^45,52–54^.

A mixed-method study in Ghana revealed that 62% of GBMSM respondents reported not testing for HIV in the past 12 months due to ISD^16,55^. Furthermore, our other previous work and others that engaged a section of YGBMSM as part of the generic GBMSM show that YGBMSM have challenges such as low HIV knowledge, ISD, and inadequate connection to care services^19,56–58^. Compared to adults, the ISD faced by YGBMSM also extends to engaging in preventative behaviors. Many cannot engage in HIV prevention conversations or be seen buying or holding a condom due to anti-premarital sex perceptions in Ghana^19,56–58^. Other studies have also reported low PrEP uptake and poor adherence to ART among this key population, with, among other things, stigma being a contributing factor^42,45^.

### The current study:

Behavioral interventions like the Many Men Many Voices (3MV) have shown efficacy in improving HIV outcome GBMSM^59–61^. Through its comprehensive approach, the 3MV intervention effectively empowers participants to make informed decisions about their sexual health, increase HIV testing rates, and adopt preventive measures such as condom use and PrEP^59^. Building upon the successes of the 3MV approach and to address the pressing need for an intervention tailored to the unique context of Ghanaian slums and the challenges faced by GBMSM, we piloted the LAFIYA HIVST intervention, which showed some effectiveness in improving HIVST among YGBMSM in slums. In this study, we seek to adapt our piloted Lafiya intervention further to incorporate a status-neutral framework, further enhancing its potential to increase HPART among GBMSM in Ghanaian slums. **LAFIYA, meaning “wellness” in Hausa,** is built upon the fundamental goal of promoting health and well-being. The LAFIYA intervention is a comprehensive program designed to address the ISD and improve HIVST among marginalized communities.

### Theoretical Underpinning

**Figure 1:**
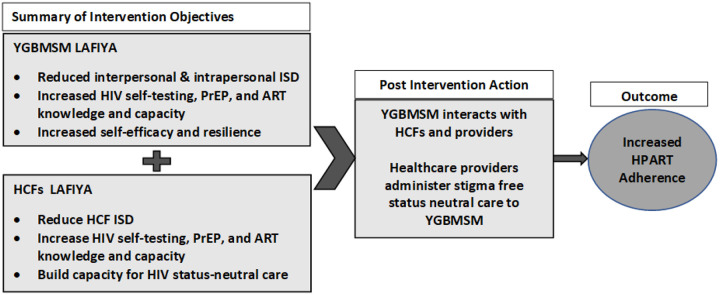
Application of the HSDF to LAFIYA intervention to increase HPART adherence

The study draws the Health Stigma and Discrimination Framework (HSDF) and scientific evidence from Ghana and SSA to postulate that stigmatized characteristics (sexual behavior, gender expression, age, and place) intersect with HIV stigma to impact HPART uptake and adherence among YGBMSM negatively^34,62–64^. The drivers and facilitators of ISD include gender norms, low HIV transmission, prevention and treatment knowledge, moral beliefs associated with HIV, and characteristics of persons (e.g., gays)^34,62–67^. ISD manifests through social exclusion, gossip, discrimination in HCFs, etc. It also manifests at the interpersonal level where YGBMSM stigmatizes other YGBMSM for gender expression or HIV status. At the intrapersonal level, YGBMSM can accept negative societal perceptions about GBMSM, PLWHIV, or slums and begin to blame themselves, develop self-doubts, etc.^34,62–64^. ISD can lead to increased risk behaviors and low HPART adherence, as internalizing the ISD forces YGBMSM to remain closeted and isolated. Thus, highlighting the importance of addressing the drivers, facilitators, and manifestations of ISD at YGBMSM and HCF levels and building the capacity at both levels (Figure1) to avert low HPART among YGBMSM^34,63,64^

#### Adapting LAFIYA, a multilevel ISD and HIV testing intervention, to a status-neutral intervention will increase HPART among GBMSM.

LAFIYA *refers* to a multilevel intervention that was developed through the adaptation of Many-Men-Many Voices (3MV). The 3MV remains the only CDC-recommended group evidenced-based intervention focused on influencing behavioral change to address the barriers to HIV prevention and care among Black GBMSM in the United States^68–71^. Our piloted LAFIYA, which is currently implemented at a larger scale through another grant, addresses Ghanaian YGBMSM contexts such as religion and ISD around HIV, sexual identity, and gender norms and expectations^2,72,73^. It also builds capacity for HIV self-testing (HIVST) by training YGBMSM on HIVST. At the HCF level, it trains selected anti-stigma nurses to offer linkage to care for YGBMSM who tested positive. Another study by members of our team also trained healthcare workers (HCWs) to offer ISD-free testing services^74^. Hence, combined, the two previously implemented interventions have begun to address ISD and HIV testing among GBMSM and HCF. But, both recruited HIV-negative GBMSM; none focused on PrEP uptake, and linkage to the care component was with selected providers (not the primary focus of the study). The proposed *LAFIYA* will address the drivers of ISD at YGBMSM and HCF levels, build a collaborative anti-stigma working relationship between GBMSM organizations and HCWs, and build capacity for 1) YGBMSM to self-initiate HPART 2) Providers to offer ISD-free status neutral HIV-care 3) Create social networks for continuous support.

## Methods

### Study design overview.

We aim to adapt and determine the preliminary efficacy of a multilevel status-neutral intervention (LAFIYA) to address HPART adherence among YGBMSM in Ghana’s slums using the following **aims. 1)** Adapt a multilevel intervention to address ISD and HPART using status neutrality among YGBMSM. **2)** Test the preliminary efficacy of the intervention to address ISD and increase HPART adherence using CRT. We will use the ADAPTT-IT framework as a guide to adapt LAFIYA^75^. The framework offers eight steps: (1) Assessment, (2) Decisions, (3) Adaptation, (4) Production, (5) Topical experts, (6) Integration, (7) Training, and (8) Testing. We will use steps 1 to 7 to implement AIM1 and the results from AIM1 to inform AIM2. Aim 1 will produce the intervention manual. AIM 2 will constitute the CRT phase, where we will assess acceptance, feasibility, and appropriateness of the intervention (**implementation outcomes**), the preliminary efficacy of the intervention to reduce ISD (**secondary outcomes**), and increase HPART adherence (**primary outcomes**) among YGBMSM.

### Study setting.

Accra, Ghana, the proposed study area, remains the primate city in Ghana, housing over 5 million people and about half of the urban population, 38% in Ghana^76–78^. Like other large cosmopolitan areas in SSA, Accra has the highest concentrations of slum communities, with half its population living in about 80 slums. About 20% of housing in Accra has an incomplete roof, yet 75% of such houses have inhabitants. About 32% of all housing in Accra consists of non-conventional structures (e.g., metal containers, wooden structures, kiosks)^78,79^. Accra also has the highest prevalence of HIV among SMM (44%) and part of the highest among the general population (3%), which is higher than the nations (2%)^80^.

### Preliminary Studies.

#### ISD experience among GBMSM and YGBMSM.

In our recent studies in Ghana^34,81^, we identified that GBMSM face various forms (e.g., experienced, anticipated, perceived, and internalized) of ISDs concerning sexual behavior or sexuality and gender expression **among their peers and healthcare workers (HCWs)**. HIV-related ISD continues to thrive due to the perception that contracting HIV signifies a punishment for sin and insufficient knowledge of HIV transmission. As such, GBMSM face insults, gossip, avoidance, and sometimes physical violence **from peers, the community, and HCWs**. In 2022, we found that over 70% of HCWs consider GBMSM mentally ill and non-Ghanaian. About 35% also said their co-workers/themselves were hesitant to care for GBMSM^81^. To circumvent ISD, many GBMSM **in slums prefer to travel outside or self-treat** as they avoid interacting HCWs, thus a need to combat ISD and increase HPART uptake. We consistently found similar results in several studies^13,34,62,64,73,74,81–89^.

#### HIV risk factors among YGBMSM.

We completed a 2022 formative study among **YGBMSM in Ghana’s slums** (Yale Fund for Lesbian and Gay Studies (FLAGS)). Qualitative interviews (n=15) show that YGBMSM have an increased HIV risk, e.g., inconsistent condom use and high transactional sex due to challenges with food insecurity. Some use sex work as their primary source of income. HIV knowledge was low, and HIV stigma was high. LAFIYA will address HIV/STD prevention knowledge gaps and reduce HIV ISD, especially age-group-specific HIV prevention needs of GBMSM youth.

#### Testing/self-testing among YGBMSM.

In 2022 (Yale Fund for Lesbian and Gay Studies (FLAGS). All interview participants (n-15) were not confident in HIV testing in slum HCFs but preferred GBMSM-friendly organizations^5^. As a follow-up, we conducted FGD with providers (n=9) who indicated that the main challenge in upscaling HIVST is the capacity to implement unassisted HIVST. In PRISM (NINR/R01NR019009), data from 10 GBMSM interviews and 8 FGDs showed: **Experiences:** 1) fear of HIV infection created a stressful HIV testing experience, and 2) friendly and supportive healthcare environment facilitated a positive experience in healthcare facilities. **Motivators or facilitators:** 1) understanding that HIV testing is an HIV prevention strategy; 2) encouragement from friends and peers; 3) understanding risk associated with sexual behaviors such as transactional sex 4) education or information on HIV; 5) access to free testing and incentives; 6) early symptoms and provider recommendation. **Barriers:** 1) negative community perceptions of HIV, 2) individual-level low-risk perception or indifference about HIV infection; 3) location and cost; 4) inadequate testing availability; 5) ISD at HCF.Thus, if we combat ISD at both HFCs and peer levels and build capacity for HPART, YGBMSM will use the services and improve their health outcomes^90–92^.

#### PrEP uptake among YGBMSM.

PrEP uptake and retention remain challenging among GBMSM Ghana**, but no PrEP intervention has been tested in the country**. Hence,we held a focus group discussion with current and previous PrEP implementing partners (7 gay and bisexual self-identified program implementers and 3 healthcare workers) to evaluate the factors affecting the initiation and continuation of PrEP. We found that **individual-level elements**, e.g., fear of PrEP side effects, the ISD associated with taking PrEP, challenges with daily medication use, and fear of HIV testing procedures, affected PrEP initiation. At **the community level**, the ISD associated with HIV impacted the interest of potential PrEP mentors to take the lead in PrEP promotion. **Implementation-level factors** include ISD from HCWs, lack of decentralized PrEP support, and target-focused implementation programs that failed to cultivate sustained PrEP understanding and uptake. Before the PrEP introduction in Ghana, FGDs conducted by our team members among GBMSM showed low knowledge of PrEP. However, once information about PrEP was provided, there was high acceptability^13^. The primary reason for acceptability was that PrEP offered extra protection against HIV. Our recent focus group under PRISM showed the same results. LAFIYA will increase education, support, and behavioral change for PrEP uptake and retention for YGBMSM instead of focusing on enrollment numbers.

**Linkage to care.** In Accra, our team conducted qualitative studies with GBMSM adolescents and adults living with HIV in 2015 (CFAR/NIH-P30 AI078498), (n=30) and 2021 (NINR/R01NR019009) (n=10). In 2015, **barriers identified include fear of being seen in HIV HCFs** and long wait times.

Motivators/facilitators included social support, HIV treatment knowledge, and positive experience from HCW^93^. In 2021, the identified barriers include community and HCF-level ISD, confidentiality issues, alternative medicine such as herbs and churches, and substance use. Motivators/facilitators included positive experiences with HCW, HIV counseling, and detailed medication information. Our 2021 quantitative analysis of 225 GBMSM living with HIV, supportive healthcare climate (OR ¼ 1.63, p < .01), vicarious HIV stigma (OR ¼ 2.73, p < .01), and age (OR ¼ 1.06, p < .004) predicted care. Conversely, HIV stigma negatively predicted care (OR ¼ 0.65, p < .05)^94^. These findings highlight the need for interventions that address linkage to care issues, especially disinformation and misinformation among GBMSM and healthcare providers. The status-neutral care will present an innovative care delivery medium, affirming and competent healthcare providers to improve care and eliminate factors (e.g., alternative medicine use and ISD.

### Aim 1: Adapt a multilevel intervention to address ISD and HPART using status neutrality among YGBMSM

#### ADAPT- ITT, Step 1 (Assessment).

We will conduct FGDs and in-depth interviews (IDI) with YGBMSM and GBMSM+NURSES providers to deepen understanding of ISD, barriers, and facilitators of HPART adherence among YGBMSM. We will integrate realities from both providers and YGBMSM to optimize the intervention potential to address ISD and increase HPART. FGDs have demonstrated suitability in studies of sexual health and capitalize on spontaneous conversational interaction in the group. IDIs have proven successful in allowing in-depth openness about perspectives on issues of a private nature^84,95,96^. We will examine the following.

#### YGBMSM.

**1)**Evaluate experiences of ISD (e.g., HIV, place, age, sexual) and HIV testing, prevention, and care and how such experiences influence HPART decisions. **2)**Assess knowledge of HIV, prevention options, willingness to test, and preferred means of ART initiation or HIV prevention after testing. **3)** Identify feasible options for engaging with testing (self-testing, home, HCF, etc.), PrEP, and ART medication, as some YGBMSM may live in shelters and face privacy challenges. **4)**Solicit recommendations for combating ISD and increasing HPART adherence. **Providers. 1)**Assess experiences with implementing HPART to YGBMSM. **2)**Assess the role of ISD and other facilitators and barriers to HPART implementation among YGBMSM. **3)**Garner updated information on implementing anti-ISD interventions in HCFs and sustainability practices over time to inform aspects of the intervention. **3)**Solicit recommendations for combating ISD and increasing HPART adherence.

### Sampling and recruitment

#### Technique: YGBMSM FGDs and IDIs:

We will combine venue and chain referral techniques to YGBMSM^97^. We have used both strategies independently in previous studies; however, integrating both will allow us to optimize reach as YGBMSM in Ghana remain hidden^34,98^. Here. The community partners will contact YGBMSM through their outreach services and invite them to participate in the study. We will then ask YGBMSM who consent to the study to refer other YGBMSM known to them. We will interview 20 YGBMSM living with HIV and include 8 to 10 persons, regardless of HIV status, in each FGD (n=5).

**GBMSM civil society organizations FGD:** we will use the purposive sampling technique to recruit 8–10 for each FGD (n=2)^99,100^. Here. We will invite participants due to their specific abilities to provide unique and in-depth information necessary to answer the research questions. This sampling method remains best for our process, considering YGBMSM in Ghana remains hidden due to ISD. The providers’ identities remain confidential and only known to the team and other GBMSM because of previous training and their roles in providing care to GBMSM in the country.

**HCF staff: A total of 6 peer-led nurses**, one from each facility, will be selected by the Ghana HIV/AIDs program to assist research assistants in recruiting staff that represents a diverse cross-section of the facility whom YGBMSM are likely to encounter during the process of HPART initiation or adherence (e.g., receptionist, security guard, nurse, HIV counselor, cashier). Each FGD (n=6) will include 8–10 persons stratified by clinical vs. non-clinical. We will secure an updated list of HCF staff and construct a random sample that will be contacted from the list while allowing staff to decline participation confidentially. The peer-led nurses will perform the liaison role throughout the study period. **Anti-ISD Trained Nurses IDIs**: We will also use the purposive sampling technique for anti-ISD nurses (n=20) who were trained as facilitators under our previous works in Ghana (LAFIYA and PRISM)

#### Sample size justification.

We are ensuring a sample size of at least 20 for each activity as it equals or exceeds other standard IDIs conducted among SSM and providers^34,98^. It’s also within the standard recommended for qualitative studies (n=19)^117^. Thus, it will allow for comprehensive coverage of necessary information to reach saturation and provide enough information to inform the next study stage.

### Inclusion and exclusion criteria.

#### YGBMSM FGD and IDI:

To be eligible for FGD, the person must be between 18 to 25 years old, currently identifies as a cis-gender man, have had sex with another man within 8 months before engaging with the recruitment team, and must reside within a slum community in the Accra metropolitan areas. To be eligible for IDIs, participants must self-disclose living with HIV. **GBMSM civil society organizations FGD.** The person must have experience providing GBMSM with HIV prevention and care services for at least one year. Must reside in Accra metropolitan area. **HCF staff** FGDs: HCF staff are eligible to participate in the FGDs if employed at a study-participating facility. **Anti-ISD Trained Nurses IDIs** HCF staff are eligible to participate if they are trained in our previous interventions and have previously delivered them in a Ghanaian HCF.

### Data collection.

#### YGBMSM.

Trained GBMSM collaborators who collected data in our previous studies with YGBMSM in Ghana will collect the data. **Providers:** Trained GBMSM+NURSE collaborators will collect provider data. The collaborators will conduct and audio record all interviews privately to ensure privacy. They will also translate interviews where the language is not English; the PI and GBMSM+NURSE team leaders will verify such translations for accuracy. For example, the PI is fluent in northern Ghanaian languages, and the team leaders are fluent in southern Ghanaian languages. We expect participants to be mixed due to the nature of slums. As such, our data collection team is combined with individuals who reflect the diversity of the participants. The recordings will then be encrypted and uploaded to the team box folder, accessible only to key personnel.

#### Data analysis.

We will use Dedoose Software to analyze the qualitative data in two phases to 1) Identify areas and content for adapting the intervention and 2) publish study findings.

**For intervention adaptation**, we will use a summative analysis process. We will form an 8-member team consisting of GBMSM+NURSES data collectors, a sample of participants, and research content experts (PI and site PI), led by a qualitative expert (Co-I, Leblanc) to review and provide critical summaries of key findings from the IDIs. Two investigators will review each transcript to ensure we establish the reliability of the information extracted and discuss conclusions and meanings of unclear messages in the team meetings. Leblanc and Abu-Ba’are will then systematically combine the summaries by identifying major points from each summary that address the drivers and manifestations of ISD, HPART experiences, options for implementing HPART, barriers and facilitators to HPART implementation, and recommendations for factors to be considered in the intervention to address ISD and upscale HPART. GBMSM+NURSES, who conducted the interviews, will review the final summaries to ensure they are consistent with their understanding and realities of the YGBMSM and HCWs interviewed. Their views will be discussed, and any corrections will be agreed upon at the team meetings to finalize the findings.

**For manuscript development**, we will conduct a more detailed qualitative content analysis. In this procedural approach, we will categorize and review data iteratively to generate conclusions based on the text’s explicit and implicit meaning. Led by Leblanc, with support from Abu-Ba’are, McMahon, Torpey, Nelson, and GBMSM+NURSES in Ghana, we will use open coding to identify categories and create a thematic codebook from the transcripts. The coders will independently apply the codebook generated to a sample of transcripts (4 each for YGBMSM and provider transcripts). They will meet to review line-by-line coding, discuss discrepancies, and review and update the codebook to ensure consistency in coding. They will then divide the remaining transcripts and independently code them using the finalized codebook. Code excerpts will then be exported from Dedoose for consolidation to answer the research questions.

#### ADAPT- ITT, Step 2 (Decision).

Using the information gathered from AIM1 (Assessment). We will form a ten-member team **(THE12**), including invited YGBMSM participants, the GBMSM+NURSES, Duure, McMahon, Torpey, Nelson, and Leblanc, led by PI, Abu-Ba’are. THE12 will meet to discuss the results and identify specific intervention activities that should be modified to address ISD and HPART. THE12 will also review to identify evidence-based best actions to address ISD and HPART among YGBMSM for consideration in our decision-making. We will then finalize the specifics of the existing intervention that needs to be adapted to ensure we fully incorporate ISD reduction and HPART/status neutrality focus on the intervention. Considering that the team has diverse persons, mostly GBMSM, with local expertise, we will ensure that the intervention content and adaptation meet the local context of law, religion, language, and HIV communication.

#### ADAPT- ITT Step 3 (Adaptation).

THE12 will conduct initial modifications of the intervention to reflect decisions in Step 2. We will then organize a 3-day mock workshop to administer and theatre test the intervention activities among a sample of YGBMSM+NURSES (n=10) who participated in the previous intervention in Ghana. During the workshop, we will implement the activities (role plays, scenarios, discussions, presentations, games, etc.) that will tackle ISD and HIVST among YGBMSM and HCF staff. As will be done in the intervention, we will implement the HCF components to nurses separately and the YGBMSM component to YGBMSM in the theatre test. We will then collect feedback from GBMSM+NURSES implementers on aspects of the intervention content and delivery that need amendments.

#### ADAPT- ITT Step 4 Production.

THE12 will collate and brainstorm about the experience in the mock intervention workshop. They will review the successful parts of the training for consolidation and devise ways to address the interventions’ challenges or less prosperous areas. We will maintain the study’s focus and be consistent with the literature on HPART, status neutrality, ISD, and relevance to YGBMSM and HCF in Ghana’s slums to ensure we produce a comprehensive yet specific manual to address ISD and increase HPART.

#### ADAPT- ITT Step 5. Topical Expert Review and Step 6. Integration.

We will provide a copy of the *LAFIYA* manual to experts on ISD, HPART, status neutrality, and YGBMSM health in Ghana and SSA for review and feedback. The experts will be asked for feedback on the consistency of the manual to address ISD and to increase HPART among YGBMSM. The experts identified include Dr. LaRon Nelson, who led several studies in Ghana and is an expert in ISD and HIV prevention and care among GBMSM. Dr. Kwasi Torpey has conducted several studies among HIV key populations and HCWs in Ghana. Francis Boakye founded Priorities on Rights and Sexual Health (PORSH), the civil-society organization partners that work with Ghanaian GBMSM and providers. Edem Zigah is a community focal person/expert in HIVST and PrEP in Ghana. **Eunice Asomani, RN, MPH, and Priscila Tawiah, RN, BSN, are anti-ISD nurses working** with PORSH and Ghana Health Service HCF, offering HIV care to GBMSM. **We will also receive feedback from the Ghana AIDS Control programme, on local program contents and consistency with local HIV prevention and care guidance**. The topical experts will give written feedback and participate in a workshop to discuss pivotal activities and modifications to the local cultural relevance and intervention implementation. THE12 will collate, review and incorporate the feedback to solidify the manual, ask follow-up questions, and discuss areas where the input is unclear to the team.

#### Step 7. Training.

In consultation with the Site PI, the GBMSM+NURSES implementers will select facilitators to deliver the intervention. **For the YGBMSM component**, the facilitators will self-identify as GBMSM and experience participating in previous HIV interventions with GBMSM in Ghana. We expect facilitators to have cultural relatability to topics and the potential anxieties and wariness of the intervention participants. The interventionists will receive a five-day training on group facilitation skills and the LAFIYA intervention from Drs Nelson and Abu-Ba’are, who have extensive experience training previous facilitators. They would be supported by Mr. Zigah and Ms. Tawiah, who are experts in HIVST, PrEP, and linkage to care; they also facilitated previous interventions among GBMSM in Ghana. **For HCF**, the facilitators will be a combination of intervention HCF staff and GBMSM selected in consultation with HCF management (Ghana health services) and CSOs. The facilitators will receive intense five-day training of trainers on the content of the curriculum and the participatory facilitation skills necessary to deliver it. They then receive five days of on-site coaching at their facilities for the first two pieces of training that they provide. Expert trainers (Dr. Abu-Ba’are and Mrs. Akumani) will train the HCW trainers. Dr. Abu-Ba’are previously coached Mrs. Akumani, who became a star trainer at her HCF and GBMSM levels in NINR’s R01NR019009. They will be supported by Mr. Zigah, Dr. Nelson, and Mrs. Tawiah, who also participated in previous training. **The team of trainers reflects the GBMSM+NURSES participatory collaboration established throughout this study**. This combination provides a feeling of safety and participant buy-in and allows the opportunity to get perspectives from the other stratum.

### STEP 8 (Testing/Aim 2): Test the preliminary efficacy of the intervention to address ISD and increase HPART adherence using CRT Design

We will pilot-test the *LAFIYA* multilevel intervention using a clustered randomized control trial (CRT) with 9 months of waitlist control. Our ultimate aim is to adapt the intervention and preliminary test it for future full-efficacy CRT ^118,119^. Although RCT is primarily used in HIV and behavioral interventions, we find CRT better due to our place-based focus and possible sample contamination. In our previous single arm-pilot (2P30MH062294 −20 // Yale #18–001890, PI, Abu-Ba’are), we identified group sleeping arrangements, observed close-knit networks of GBMSM, and the communal nature of slums as contaminants. We will create two pairs of facilities matched by size and location in each city and randomly assign them to receive the LAFIYA-HCF intervention or the control group. Based on friendship networks, we will create GBMSM groups of 5 to 8 members and randomly assign YGBMSM groups to the LAFIYA-YGBMSM intervention or control group. YGBMSM in the intervention group will be matched with intervention HCF for status-neutral services. The YGBMSM in the waitlist control group will be matched with HCFs in the waitlist control for regular testing, PrEP, and care services. Nurses from the intervention HCF will participate as guest facilitators in one of the LAFIYA-GBMSM sessions. They will use that opportunity to contact YGBMSM to visit their facility for status-neutral care. We will collect 3, 6, and 9 months post-intervention data to measure the preliminary efficacy of the intervention and then implement the intervention in the waitlist group after the completion of data collection. We will use 9 months instead of 6 to allow ample time to measure multiple components, **such as viral suppression,** over a sufficient time. **Our primary hypothesis** is that LAFIYA will increase adherence to HPART among YGBMSM participants. **Our secondary hypothesis** is that the multilevel intervention will reduce the ISD that HCF staff convey and YGBMSM experience in HCFs. It will build the HCF’s capacity to implement status neutrality. We will then **conduct a follow-up qualitative study** (FGDs and IDIs) post-intervention among implementers and participants to evaluate their experiences with the intervention and to seek a more profound understanding or explanations for some of the survey findings.

### Sampling and recruitment

#### HCF.

We will target training all clinical and non-clinical HCF staff who may encounter YGBMSM seeking HPART services at the facility (e.g., nurses, lab staff, guards, receptionists, etc.). The GBMSM+NURSES trainers and peer-led nurses will work closely with facility management to select participants and coordinate training attendance and data collection logistics to minimize service disruption. The training will be held on-site and fitted into the facility activities as done in our previous works. Based on our preliminary analysis of reported numbers from the six facilities, and prior studies, we estimate about 1500 participants across sites. In our earlier studies, 60 to 70% of the staff was trained, but GBMSM+NURSES partners reported incidents of ISD among other staff who were not trained. For the surveys (n=300), we will secure a list of intervention participants from various departments, stratify participants (clinical and non-clinical) and select participants using simple random sampling from each stratum. **For the follow-up** FGDs (n=5), we will use the process and criteria defined in **AIM1**, with 8 to 10 persons per FGD and 20 anti-stigma nurse trainers for the IDIs. **YGBMSM**. We will use a combination of venue-based and snowball sampling to recruit YGBMSM (n=160) per site (totaling 320). Like starfish sampling, this process overcomes the weaknesses of using only venue-based or peer-driven recruitment. The implementing partners will invite known YGBMSM that meets the criteria for the CRT. We will then select initial seeds among the YGBMSM and ask them to invite their peers to the study using coupons. This chain-referral process will continue until we reach the number of participants needed. Based on the referral chains, we will use the seeds and nodes to identify peer groups of 5 to 8. **For the follow-up** FGDs (n=5), we will use the process and criteria defined in **AIM1**, with 8 to 10 persons per FGD and 20 YGBMSM living with HIV for the IDIs.

### Power.

#### YGBMSM:

We perform the power analysis to detect the difference in intervention outcomes using a sample measurement scale (HIV-ASES)^101^ between intervention and control (n=240, control arm=120, intervention arm = 120) by adjusting the Intraclass correlation coefficient (ICC) for the friend group. In a similar setting, a network membership explained 4.08% (i.e., ICC=0.04) of the total variance in outcomes^102^. The ICC of 0.04 and 8 individuals in a friend group will produce the design effect (DE) of 1.28. Approximately 15 peer groups will be randomly assigned to each study arm. A DE of 1.28 and a sample of 240 (120 per arm) will have 92.7% power to detect the medium effect size of 0.50 at a 5% significance level. The medium effect size of 0.50 implies a 5.6 point (SD=11.2) on the outcomes and 0.62 points (SD=1.23) based on standard deviations from HTAS. The sample size of 120 in the intervention group will have 99% and 82% powers to detect the medium and small effect sizes of 0.50 and 0.30, respectively, on the outcome changes within the group. With a 10% attrition rate, 216 participants will have 90% power to detect the medium effect size of 0.50, and 108 participants receiving the intervention will still have 99% for the medium effect size after adjusting for ICC of 0.04.

#### HCF:

We anticipate 300 workers from 6 facilities. Assuming the minimum number of 30 workers in each facility and ICC of 0.01, a design effect of 1.36 is expected. We anticipate 96% and 84% powers to detect the medium effect sizes of 0.5 and 0.4 for the difference in outcomes between the two interventions. We expect the proposed sample sizes of YGBMSM and HCWs to be enough to detect the medium effect sizes of the intervention.

### Implementation of the intervention

#### Implementation procedure.

The facilitators trained in aim one will receive a copy of the manual on delivering each component of the adapted intervention. The intervention will be participatory.

#### YGBMSM:

The implementation partners will recruit the YGBMSM to attend a 3-day weekend retreat at a location they identified with support from the site PI and Contact PI. Considering that YGBMSM remain hidden, we expect to utilize places we used in previous interventions^3,5,8,9,33^. Such locations have already been appraised and assured security. YGBMSM, who accept to participate in the intervention, will receive retreat dates. Participants will attend an orientation to obtain information about the retreat’s logistics and format and establish ground rules for appropriate behavior.

#### Criteria:

The person must be between 18 to 25 years old, currently identifies as a cis-gender man, have had sex with another man within 8 months before engaging with the recruitment team, and must reside within a slum in the Accra metropolitan area. **Randomization**. When we complete the recruitment and baseline survey, we will stratify peer groups by the number of peer members and give a group ID to each peer group. Within each stratum with an equal number of peers, we will randomly select groups for intervention using simple random sampling. Thus, we will keep equal samples and peer groups between the two study arms.

#### HCF:

The participatory exercises in this intervention will be delivered over two days to groups of 15 to 25 HCWs mixed by department and level (clinical/non-clinical) arranged based on the schedule of workers to ensure facility services are not disrupted.The team will work with management to assign people to the intervention before or after their shift. We used this process in our previous studies and successfully implemented the testing intervention across 8 facilities and over 1000 participants^34,73,74,81,98^

#### Randomization.

We will assign 3 HCFs to intervention or control groups based on size; we will first stratify 6 HCFs into groups of two, larger, medium, and smaller sizes, and assign one from each stratum to either arm using SRS.

### Appraisal of intervention.

#### Data collection.

Participants will complete baseline, 3, 6, and 9 months post-intervention surveys. Trained GBMSM+NURSE partners who collected surveys in our previous studies will conduct the surveys^34,73,74,81,98^. **For YGBMSM**, the data collection location will be at the designated sites (e.g., office of community partners) determined as safe for YGBMSM in Ghana due to their extensive experience working with GBMSM on the grounds in Ghana. **For HCFs**, the data collectors will visit the facilities led by the team leader at the facility to hand over the surveys to providers, who will self-complete them at their convenience. **For PrEP and ART** adherence **biomarkers**, the HCF will provide them with consent from participants during routine check-up visits.

### Measures.

#### Demography, HIV testing, and ISD descriptive variables.

We will assess important baseline characteristics for describing the sample, including age, income, gender, education, ethnicity, relationship status, religion, sexual orientation, sexual behavior, HIV knowledge, HIV testing, PrEP and ART readiness, and history. Experiences of ISDs, access, and utilization of health and social services, civic engagement, and neighborhood. **Primary outcomes:** At baseline, 3, 6, and 9 months, we will measure the preliminary efficacy of LAFIYA. We will measure knowledge of HIV and HPART; and willingness to adhere to HPART pre and post-intervention. HPART adherence characteristics (rates, time, mode, location) and biomarkers (Table 2). **Secondary outcomes:** For ISD reduction, we will measure internalized and interpersonal level ISD at baseline and post-intervention surveys among YGBMSM. We will measure HCF-level ISD to understand participant experiences at baseline and changes in HCW stigmatizing beliefs and practices (Table 2). **Implementation outcomes:** We will measure acceptability, appropriateness, and feasibility using expanded Proctors validated measures at pre-immediate and post-intervention surveys at all levels (Table 2)^103^.

#### Follow up FGDs and IDI.

We will **conduct a follow-up qualitative study** (FGDs and IDIs) post-intervention among implementers and participants to evaluate their intervention experiences and seek a more profound understanding or explanation for some of the survey findings. We will seek to know YGBMSM’s experiences with participating in the intervention, HPART experiences, and factors that remain a barrier to HPART despite enrolling in the study. We will seek to understand HCW experiences with delivering or taking part in the intervention and how that has translated to improved service delivery or otherwise in the HCF. CSO providers will also explain their experiences and recommendations for improving the intervention for a scale-up.

#### Data analysis.

We will use the intent-to-treat (ITT) technique to analyze data. We considered other methods (e.g., per protocol approach), but ITT is less biased since all participants are assessed whether they received the intervention or not. Data collected will be exported to Stata for cleaning and further analysis. Using the Monte Carlo Markov Chain approach based on a multivariate normal distribution of items, we will assess missing patterns in the instrument and impute missing responses with at least 70% of responses. Instruments scores will be calculated with observed and imputed responses. We will summarize the characteristics of participants and calculate Cronbach’s alpha to check internal reliability within scales. We will calculate a mean with a confidence interval for the variables and proportions of responses to the primary and secondary outcome variables. Variables with skewed distribution will be considered for being log-transformed or categorized in ordinal form. We will run inferential statistics such as paired t-test to compare pre-and post-means, McNamara’s test to compare pre-and post-proportions, and correlation analysis to examine the relationship between variables. We will also develop longitudinal models of the outcomes repeatedly measured at baseline, 3, 6, and 9 months post-intervention using a generalized linear mixed effect model (GLMM) with random intercept and peer group, which incorporate ICCs. The GLMM will include time-group interaction to estimate the intervention effect and pre-post changes. The GLMM will consist of all completed observations and drop-outs observed at least once post-intervention. Thus, we will not miss any individuals unless they are entirely missing at all post-intervention periods. Residuals will be assessed for normality assumption and influential outliers diagnosed with leverage, Cook’s distance, and DFFIT. The GLMM will be replaced with logistic and Poisson regressions with random effects when an outcome is binary and count data. We will repeat the same models separately for each site and compare the effect sizes between the sites. When we find any demographics, which are not equivalent between the two interventions, they will be included in the model as covariates. Acceptability, feasibility, and appropriateness will be evaluated with descriptive statistics of the implementation outcomes. **The process defined in AIM**1 will be used to analyze the follow-up qualitative data.

#### Potential challenges and solutions.

**Retention of participants.** Since we will be working with young adults and socially vulnerable persons, there is a high possibility that some of them will be mobile. As such, we will ensure we invite participants who are sure of their availability during the study period. We will also follow up periodically to check on their plans, and device means to ensure continuity. **Safety and security.** Although safety issues can affect retention, we have successfully established structures for all components of our studies that have provided participant security for over a decade^34,73,74,81,98^. Our partner organizations have activist and legal connections in place for externalities. We also get support from Ghana’s health and security services in case of external threats (we did not have to activate these security and legal structures as we have not encountered external threats in the previous works.

#### Strengths and future directions.

Our experience working in Ghana shows that due to ISD and fear of disclosure, GBMSM living with HIV hide their status to join studies if HIV-negative peers are recruited. Many GBMSM default and do not go for follow-up testing since that has been the primary focus of all previous studies in the country. The status-neutral approach eliminates both challenges and will allow us to address ISD while increasing HPART uptake. **We have a strong team with significant institutional backing from Ghana Health Services and HIV policymakers, thus providing an opportunity to understand YGBMSM’s lived experiences and factors at the individual, peer, geographical, and HCF levels affecting** HPART practices, awareness, and willingness to participate. The findings can inform future studies and interventions to address HIV prevention and care among YGBMSM within similar sociocultural contexts in Africa and elsewhere. Our current focus on HPART aligns with Ghana’s new commitment stipulated in its 2019 National HIV and AIDS Policy to increase HPART among key populations. **Future directions:** We will use the findings to finalize an efficacy intervention trial protocol to improve HPART among YGBMSM in Slums.

#### Ethical considerations.

This study will undergo review and approval by the Ghana Health Service Ethics Review Committee. Any modifications to the protocol will necessitate an approved amendment from the **Ethics Review Committee (ERC)** prior to implementation. Once all research activities have concluded, a study closure report will be submitted to the IRB. Additionally, any other study-related events such as data breaches or protocol deviations will also be promptly reported to the IRB.

#### Process of consent/Assent.

The informed consent process for this study will be conducted as follows:

- Research assistants from partner organizations will administer the interviews and surveys, providing participants with the option to self-complete the survey in person.
- Detailed consent forms, approved by the ethics review committee, will be provided to participants, outlining the study questions, procedures, and potential risks involved. Prior to initiating any study intervention or procedures, written documentation of informed consent will be obtained from participants.
- Participants will be given the opportunity to review the consent forms themselves or have them read aloud by the interviewer. The research study will be explained to participants, allowing them to ask any questions they may have. This discussion will take place **in a private room at a secure location provided by our community partner organizations. Written consent will be obtained**.
- Participants will be given sufficient time to carefully review the consent form, ask questions, and discuss the study with their family members or surrogates before making a decision to participate.
- It will be clearly communicated to participants that their participation is voluntary and they have the right to withdraw from the study at any time without facing any negative consequences. A copy of the informed consent document will be provided to participants for their own records.

#### Confidentiality and security of data.

Participants’ confidentiality will be safeguarded to the utmost extent by the researchers. Neither the research team nor any partner organization will mention participants by name in any reports. To ensure confidentiality, the data collected from participants will be assigned unique codes for identification purposes. Participants’ personal information, including contact details, will be stored separately from other study information in order to maintain confidentiality. Participants will not be identified by their actual names, but rather by titles such as “key informants” or “providers,” or by their assigned participant IDs. Ghana Health Service Ethical Review Committee (GHS-ERC) may have access to the research data as part of its routine monitoring duties. This access by GHS ERC is vital to ensure the study complies with ethical and regulatory standards and provides an additional layer of oversight to maintain the ethical integrity of the research. This will be duly communicated to the study participants.

Data storage and access. The security and confidentiality of all questionnaires and data collection tools. After data capture, these materials will be stored in a locked file cabinet to prevent unauthorized access. Additionally, all data files entered into the computer will be protected with passwords, adding an extra layer of security to prevent unauthorized viewing or manipulation. Data will be stored for a period of five years after the conclusion of the study. Subsequently, the data will be securely destroyed in accordance with research data retention guidelines, ensuring that confidentiality and privacy are maintained over the long term. This data retention period complies with standard practices for research data management and allows for potential data verification, replication

#### Data and safety monitoring plan.

The researchers will take every measure to protect the confidentiality of the participants. No identifying information, such as names, will be included in any reports by the research team or partner organizations. Instead, unique codes will be used to identify the data collected from participants. Personal information, including contact details, will be kept separate from other study information to maintain confidentiality. Participants will be referred to by titles such as “key informants” or “providers,” or by their assigned participant IDs, rather than their actual names. As part of its routine monitoring duties, the Ghana Health Service Ethical Review Committee (GHS-ERC) may obtain access to the research data. This access is crucial for ensuring that the study adheres to ethical and regulatory guidelines, and provides an extra level of oversight to maintain the ethical integrity of the research. The study participants will be notified of this accordingly.

#### Reporting of adverse events.

The study has been carefully planned to reduce any potential risks, and is closely monitored by the MPIs. The MPIs are responsible for maintaining the accuracy of the data and conducting regular safety assessments. If any concerns arise, the MPIs have the power to make necessary adjustments, halt enrollment, or terminate the study if necessary. Furthermore, the (GHS-ERC) are responsible for providing additional oversight and assistance to ensure that all protocols are followed and that all participants are safe.

## Discussion

By strategically combining elements of LAFIYA and the 3MV intervention within a status-neutral framework, we envision a comprehensive and innovative approach that not only increases HIV testing, PrEP, and ART uptake but also empowers GBMSM to take control of their sexual health. Through this adapted intervention, we aspire to make significant strides in reducing HIV incidence and improving overall health outcomes among GBMSM in Ghanaian slums, ultimately contributing to the broader goal of achieving health equity for all. We aim to adapt and determine the preliminary efficacy of a multilevel status-neutral intervention (LAFIYA) to address HPART adherence among YGBMSM in Ghana’s slums using the followingaims:1. Adapt a multilevel intervention to address ISD and HPART using status neutrality among YGBMSM, and 2. Test the preliminary efficacy of the intervention to address ISD and increase HPART adherence using CRT.

### Trail Status

This study was registered on clinicalTrail.gov, with identifier number NCT06312514 on 03/14/2023. Recruitment has not begun for this study.
